# Synthetic circuits based on split Cas9 to detect cellular events

**DOI:** 10.1038/s41598-023-41367-z

**Published:** 2023-09-11

**Authors:** Alicja Przybyszewska-Podstawka, Jakub Czapiński, Joanna Kałafut, Adolfo Rivero-Müller

**Affiliations:** Department of Biochemistry and Molecular Biology, Medical University of Lublin, 20-093 Lublin, Poland

**Keywords:** Synthetic biology, Genetic engineering

## Abstract

Synthetic biology involves the engineering of logic circuit gates that process different inputs to produce specific outputs, enabling the creation or control of biological functions. While CRISPR has become the tool of choice in molecular biology due to its RNA-guided targetability to other nucleic acids, it has not been frequently applied to logic gates beyond those controlling the guide RNA (gRNA). In this study, we present an adaptation of split Cas9 to generate logic gates capable of sensing biological events, leveraging a Cas9 reporter (EGxxFP) to detect occurrences such as cancer cell origin, epithelial to mesenchymal transition (EMT), and cell–cell fusion. First, we positioned the complementing halves of split Cas9 under different promoters—one specific to cancer cells of epithelial origin (_*p*_*hCEA*) and the other a universal promoter. The use of self-assembling inteins facilitated the reconstitution of the Cas9 halves. Consequently, only cancer cells with an epithelial origin activated the reporter, exhibiting green fluorescence. Subsequently, we explored whether this system could detect biological processes such as epithelial to mesenchymal transition (EMT). To achieve this, we designed a logic gate where one half of Cas9 is expressed under the _*p*_*hCEA*, while the other is activated by TWIST1. The results showed that cells undergoing EMT effectively activated the reporter. Next, we combined the two inputs (epithelial origin and EMT) to create a new logic gate, where only cancer epithelial cells undergoing EMT activated the reporter. Lastly, we applied the split-Cas9 logic gate as a sensor of cell–cell fusion, both in induced and naturally occurring scenarios. Each cell type expressed one half of split Cas9, and the induction of fusion resulted in the appearance of multinucleated syncytia and the fluorescent reporter. The simplicity of the split Cas9 system presented here allows for its integration into various cellular processes, not only as a sensor but also as an actuator.

## Introduction

In synthetic biology, most of the logic control developed so far employ transcriptional regulators e.g., transcriptional activators or repressors to regulate gene expression^1–5^ to either increase reaction rates in metabolic pathways or respond to new input signals. While these genetic circuits work very predictably in prokaryotes, in eukaryotes they often require the use of genetic elements from prokaryotes or simple eukaryotes.

The domestication of CRISPR systems, the prokaryotic “immune system” that confers resistance and memory to phageal genetic elements, to eukaryotic cells has opened a new level of genetic engineering. CRISPR/Cas9 is composed by Cas9 endonuclease, responsible for executing the desired genetic modifications, and two small RNA molecules named CRISPR RNA (crRNA) and trans-activating CRISPR RNA (tracrRNA). While crRNA contains targeting sequence, the tracrRNA acts as a scaffold for Cas9 endonuclease^6^. In order to simplify the system, the crRNA and tracrRNA have been fused into a single guide-RNA (gRNA)^7–9^. Cas9/gRNA generates double-strand DNA breaks which can be repaired by either nonhomologous end-joining pathway (NHEJ), causing errors such as insertion/deletions (indels), or by homology-directed repair (HDR), which can be used to introduce specific DNA sequence changes. Such simplicity of CRISPR systems has made them the most adaptable system to manipulate endogenous genetic information, from gene deletion (knockout)^10–13^, gene insertion (knock-ins)^14–16^, to gene regulation (CRISPRi and CRISPR-TA)^17^, and genome editing^18–20^.

The Cas9 system has also been integrated into genetic logic gates whereas the input is provided by gRNAs that respond to a particular stimulus, such as the presence of a specific chemical^21,22^ or the activation of a specific signaling pathway or regulate multiplexed circuits to control transcriptional regulation (activation/inhibition) by modified Cas9 forms (CRISPRa and CRISPRi, respectively)^23–26^.

Yet, CRISPR/Cas9 has been rarely used as a sensor for biological events i.e., for identification of cancer cells^27^ or to control a complex phenotype such as cell fate^28^. In these cases, Cas9 and the gRNA have been positioned under specific promoters, each becoming one input to the AND logic gate. A split Cas9 system has previously been reported although not applied into logic circuits^29^, although it has been used to generate drug- and light-inducible systems by fusing halves of Cas9 to hetrodimerizers^30,31^.

The application of Cas9 into logic gates remains relatively unexplored, given its functionality both an actuator and a “memory” by permanent modifications on DNA. Here, we present another use of CRISPR/Cas9 by creating a synthetic (AND) logic gate using self-assembling split Cas9 (named Cas4.5_N_ and Cas4,5_C_—as they sum up to Cas9), facilitated by DnaE inteins^32^, that need to be co-expressed (input 1 and 2) to activate a reporter, leading to a specific output (outcome). While studying cellular events can be detected by biochemical assays or biosensors, there are very few examples on the use of synthetic logic gates to record cellular events in eukaryotic cells.

Moreover, the presented 2-input system results in an active actuator that can be applied to many other biological outcomes. We show that detection of cell origin, phenotypical transition or cell–cell fusion can be specifically detected by split Cas9 logic gates.

## Experimental section

### Materials and reagents

Plasmid pSpCas9(BB)-2A-Puro (PX459) V2.0 was a gift from Feng Zhang (Addgene plasmid #62988^33^), pU6-sgGFP-NT1 was a gift from Stanley Qi & Jonathan Weissman (Addgene plasmid #46914; http://n2t.net/addgene:46914; RRID:Addgene_46914)^8^), pCAG-EGxxFP-*Cetn1* was a gift from Masahito Ikawa (Addgene plasmid #50717; http://n2t.net/addgene:50717;RRID:Addgene_50717^34^), AmCyan-P2A-mCherry was previously created by us^35^ (Addgene plasmid #45350; http://n2t.net/addgene:45350; RRID:Addgene_45350^35^). The TWIST1 binding domain (*TBD*) promoter was created previously by us^36^, DnaE intein plasmids were a gift from Hideo Iwai (Addgene plasmid #34549; http://n2t.net/addgene:34549; RRID:Addgene_34549^37^ and #15335; http://n2t.net/addgene:15335; RRID:Addgene_15335^38^). Cell lines HEK-293, HSF, CCD 841 CoTr, H1299, H2170, SW480, A375, HeLa, U-2 OS, C2C12 and GC-2 were obtained from ATCC; H2170 stable cell line with vimentin reporter (VRCs) was created by us^39^. KOD-Xtreme hot-start DNA polymerase (Merck Millipore), DreamTaq™ Green PCR Master Mix (ThermoFisher Scientific), endonuclease *DpnI* and *BbsI* (Thermo Fisher Scientific), T7 Ligase (Thermo Fisher Scientific) and PNK (Thermo Fisher Scientific), Polyethylene Glycol Hybri-Max 1450 (PEG) (Sigma-Aldrich), TGFβ (human TGFβ1, Biorbyt, Cambridge, United Kingdom), Gibson Assembly® Master Mix (NEB), Ampicillin (BRAND), DNA Clean and Concentrator and Zyppy Plasmid Kits (Zymoresearch), Hoechst 33342 (Cayman), Dulbecco’s Modified Eagle Medium (DMEM/F-12), Dulbecco’s Modified Eagle Medium—high glucose (DMEM) (Sigma Aldrich), Penicillin and Streptomycin (Sigma Aldrich), fetal bovine serum (Sigma Aldrich), horse serum (Sigma Aldrich), and geneticin G418 (ThermoFisher Scientific). All primers were bought from Genomed (Warsaw, Poland).

### PCR

All primers were designed in SnapGene Viewer and purchased from Genomed (Warsaw, Poland), Syncytin1 was recoded based on the amino acid sequence from the The Human Protein Atlas and ordered as a gBlock (ThermoFisher). PCR reactions were performed using a high-fidelity polymerase (KOD-Xtreme, Merck Millipore) according to manufacturer protocol. All products used for molecular cloning were then digested with fast digest *DpnI* restriction endonuclease (Thermo Scientific) to get rid of the methylated template plasmid. PCR products were column-purified before cloning using DNA Clean & Concentrator (Zymo research).

### Molecular cloning

All vectors were created using Gibson Assembly^40^ according to manufacturer protocol (NEB). In brief, inserts and vectors were amplified by PCR, or digested using restriction enzymes in selected cases, followed by gel-purification and concentrations measured. Appropriate amounts were used for Gibson reaction (ratio insert:vector 3:1). Then, electroporation with 2ul of the reaction was performed by using prepared electrocompetent DH10B *E. coli* bacteria, and after 45 min, bacteria were plated onto agar plates for selection with an antibiotic. A screening colony was taken using PCR, and the sequence was verified. The EGxxFP-*Cetn1* plasmid was modified by inserting the Puro-T2A cassette from pU6-sgGFP-NT1 (Addgene plasmid #46914) by recombination resulting in the Puro-T2A-EgxxFP plasmid. Cas4.5_N_ was amplified by PCR from Cas9 plasmid and then fused at its *C*-terminus to DnaE_N_ intein, while Cas4.5_C_ was fused at its *N*-terminus to DnaE_C_ intein. In the next step, the CMV general promotor (*pCMV*) in Cas4.5N-DnaE_N_ was replaced by *hCEA* or the TWIST1 binding domain *(TBD)* specific promoters/genetic elements. Resulting vectors: _p_hCEA-Cas4.5_N_-DnaE_N_, TBD-Cas4.5_N_-DnaE_N_, TBD-mCherry and _p_CMV-Cas4.5_C_-DnaE_C_. The gRNAs against EGxxFP-*Cetn1* was cloned into both the Cas9 and the Cas4.5_C_ plasmids, following the Zhang’s lab protocol^33^ using T7 ligase and *BbsI* (both from ThermoFisher).

### Cell culture and transfection

Human embryonic kidney 293 (HEK293) cells, human skin fibroblast cells (HSF), human normal colon epithelial cells (CCD 841 CoTr), lung cancer cell lines (H1299, H2170, H2170 VRCs), malignant melanoma cell line (A375), cervical carcinoma (HeLa), colon cancer cell line (SW480), human bone osteosarcoma cell line (U-2 OS), murine myoblasts (C2C12), and murine spermatocyte cell line (GC-2), were cultured under standard conditions in the incubator (37 °C/5% CO_2_). Media DMEM/F12 (Sigma Aldrich) were supplemented with 10% Fetal Bovine Serum (PromoCell) and 1% Penicillin/Streptomycin solution. Transfections have been carried out using TurboFect reagent (Thermo Fisher), and Lipofectamine 3000®. Ratio DNA/reagent have been optimized for each cell line in order to obtain the best transfection efficiency.

One day before transfection H1299 and H2170 VRCs were seeded at density 5 × 10^4^ cells/well into a 24-well plate, and co-transfected with one half of Cas9 protein (_p_CMV-Cas4.5_C_) driven by general promoter, construct with EGxxFP reporter plasmid, *Cetn1* gRNA and second half of Cas9 (TBD-Cas4.5_N_) under a promoter activated during EMT—under the control of TWIST1, using Turbofect™ transfection reagent following the manufacturer’s protocol. Transfection with only one half of Cas9 protein was used as a negative control (NC). Next day after transfection cells were exposed to TGFβ (10 ng/mL) for 72 h to evaluate the expression level of EMT-related gene *TWIST1*. All experiments were performed in at least triplicate.

Equally, 24 h before transfection HSF, CCD 841 CoTr, C2C12, GC-2, A375, SW480, H1299, HeLa and U-2 OS cells were seeded at density 5 × 10^4^ cells/well into a 24-well plate, and co-transfected with one half of Cas9 protein (_p_CMV-Cas4.5_C_) driven by general promoter, construct with EGxxFP reporter plasmid, *Cetn1* gRNA and second half of Cas9 (_*p*_*hCEA-Cas4.5*_*N*_) under an epithelial cancer-specific promoter, using Lipofectamine 3000® (CCD 841 CoTr, A375) or Turbofect™ (H1299, SW480 and HeLa) transfection reagents following the manufacturer’s protocol. Transfection with only one half of Cas9 protein was used as a negative control (NC), and transfection contains mCherry was used as a transfection control. All experiments were performed in at least triplicate.

Cell lines HEK293, H1299, and H2170 were seeded at density 5 × 10^4^ cells/well into a 24-well plate 24h before transfection. Using Turbofect™ transfection reagent following the manufacturer’s protocol, cells were transfected with either one half Cas4.5_N_ with EGxxFP reporter plasmid, *Cetn1* gRNA, or the second halve _p_CMV-Cas4.5_C_ with Syncytin1 plasmid. Transfection with only one half of Cas9 protein was a negative control (NC). In PEG treatment, cells were transfected with either Cas4,5, gRNA and reporter plasmids, and next day after transfection cells were exposed to 50% solution of PEG for 3 min at RT to induce cell–cell fusion. Similarly, to create a stable cell lines HEK293, H1299, and H2170 cells were seeded at density 5 × 10^4^ cells/well into a 24-well plate one day before transfection. Using Turbofect™ transfection reagent following the manufacturer’s protocol, cells were transfected with each halves (_p_CMV-Cas4.5_N_; _p_CMV-Cas4.5_C_). After 48 h, cells were selected with Puromycin (0.75–1 mg/ml) for over three weeks to generate stable lines. Then, stable cell lines were co-transfected—one half Cas4.5_N_ with EGxxFP reporter plasmid, *Cetn1* gRNA, and second halve _p_CMV-Cas4.5_C_ with Syncytin1 plasmid.

### C2C12 differentiation

For ddifferentiation of mouse myoblast cells (C2C12) general media was replaced with differentiation media (DMEM high glucose without sodium pyruvate, 2% horse serum, and 1% Penicillin/Streptomycin). Then, cells were seeded on 24-well plate at 90% confluency. Cells started to align to each other and fuse into multinucleated myotubes after 48 h. Medium was changed every 48h.

### Wound-healing assay

For the Wound-healing assay cell lines of H1299 and H2170 VRCs (vimentin reporter cells)^39^ were seeded into 12-well plates at a density 5 × 10^4^ cells/mL. One day later were co-transfected with one half of Cas9 protein (_p_CMV-Cas4.5_C_) driven by general promoter, construct with EGxxFP reporter plasmid, *Cetn1* gRNA and second half of Cas9 TBD-Cas4.5_N_ under the specific promoter activated during EMT—under the control of TWIST1, resulted in *EGFP* expression. 24 h after transfection cells were exposed to TGFβ (10 ng/mL) for 72 h to evaluate the expression level of EMT-related gene *TWIST1*. Next day the monolayer of cells was scratched to form a linear wound. The Wound-healing Assay images were performed up to 72h under Nikon T*i* Eclipse confocal microscope.

### Electrofusion

For cellular fusion was used the BTX’s ECM 380 ElectroCellManipulator. Condition were optimized, two settings of device voltage was used and of controlled electrical pulse: 700 V for 15 µs (3 pulses) and 300 V for 500 µs (1 pulse), acting on the cells. For cell electroporation was used hypotonic medium. After experiment cells were resuspended in nutrition medium.

### Confocal microscopy

The cells were cultured on glass bottom plates suitable for confocal imaging. Visualization has been carried out using Nikon T*i* Eclipse microscopy. The nuclei of cells before imaging have been Hoechst 33342 stained (concentration 10 µg/mL).

## Results

Naturally split DnaE intein (DnaE_N_ and DnaE_C_) has high self-affinity followed by efficient splicing and ligation of the flanking exteins^38^ but only when DnaE_N_ ends with a Cysteine (Cys;C), while DnaE_C_ begins with the following di-amino acids: Cys followed by either Tyr (CY), Trp (CW), Phe (CF), His (CH) or Met (CM)^41^. After analyzing the Cas9 amino acid sequence and we found only one CY [amino acids (aa) 119–120] and one CF (aa 613–614) (sequences in Supplementary material S1). Since the latter is close to the middle, it was selected, and the two resulting halves have been termed: Cas4.5_N_ (aa 1–613) and Cas4.5_C_ (aa 613–1423).

Inteins can spontaneously self-assemble and induce protein ligation of their fused cargoes—in this case, the complementary Cas4.5 fragments, generating the holo-Cas9 endonuclease.

**Figure 1:**
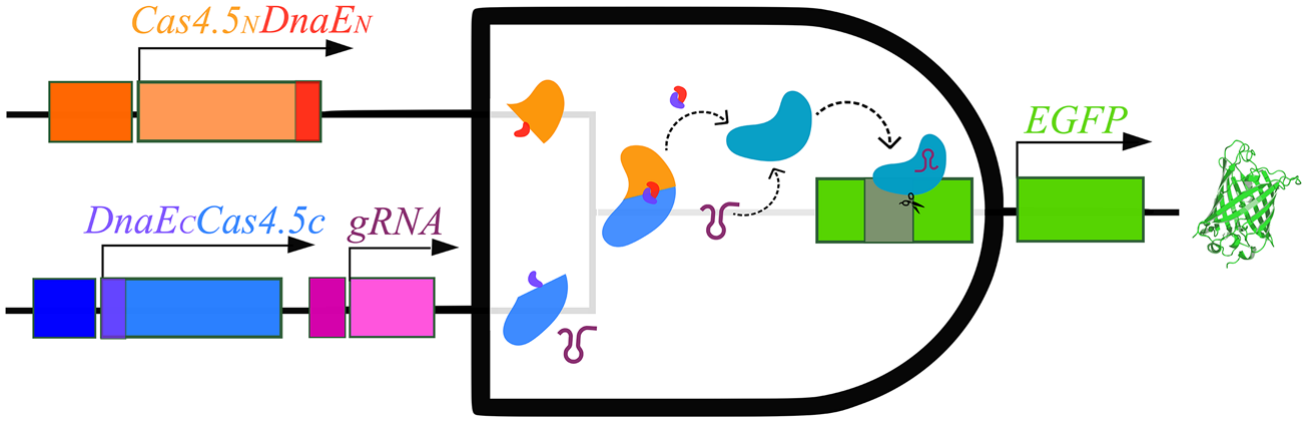
Scheme of the principle of split-Cas9 as an AND logic gate.

To detect Cas9 activity we adopted and modified the EGxxFP reporter^34^. This reporter is constructed on the basis of truncated but overlapping regions of the enhanced green fluorescent protein (*EGFP*) gene, with an early stop codon after 2/3 of the coding sequence, followed by a genomic (non-coding) fragment from the *Cetn1* gene (stuffer) and a second fragment of the fluorescent protein covering about 500 bp of homology and the missing 1/3 of the respective gene. In the presence of active Cas9 and a targeting gRNA to the stuffer region, the homology regions undergo homologous recombination, creating the full-length *EGFP* gene and the fluorescent protein being expressed (Fig. 1). Co-expression of the 2 halves of Cas9 lacking inteins do not form a holo-enzyme as no reporter activity is detected (not shown).

Expression of individual Cas4.5 halves in cells, together with the gRNA, showed no reporter activity, proving that they are innocuous on their own. However, when both halves, containing inteins, were expressed in the same cell, Cas9 was active as detected by recombination of the reporter (EGxxFP → EGFP).

We then created an AND logic gate where one input is under a conditional promoter, while the other halve is under the ubiquitous (_*p*_*CMV*) promoter (Fig. 1). Certain parts of the system depend on specific promoters and other genetic elements for well-defined subpopulations. Based on that, we are able to program in what kind of cells the system will be activated.

### Cell origin sensing system

**Figure 2:**
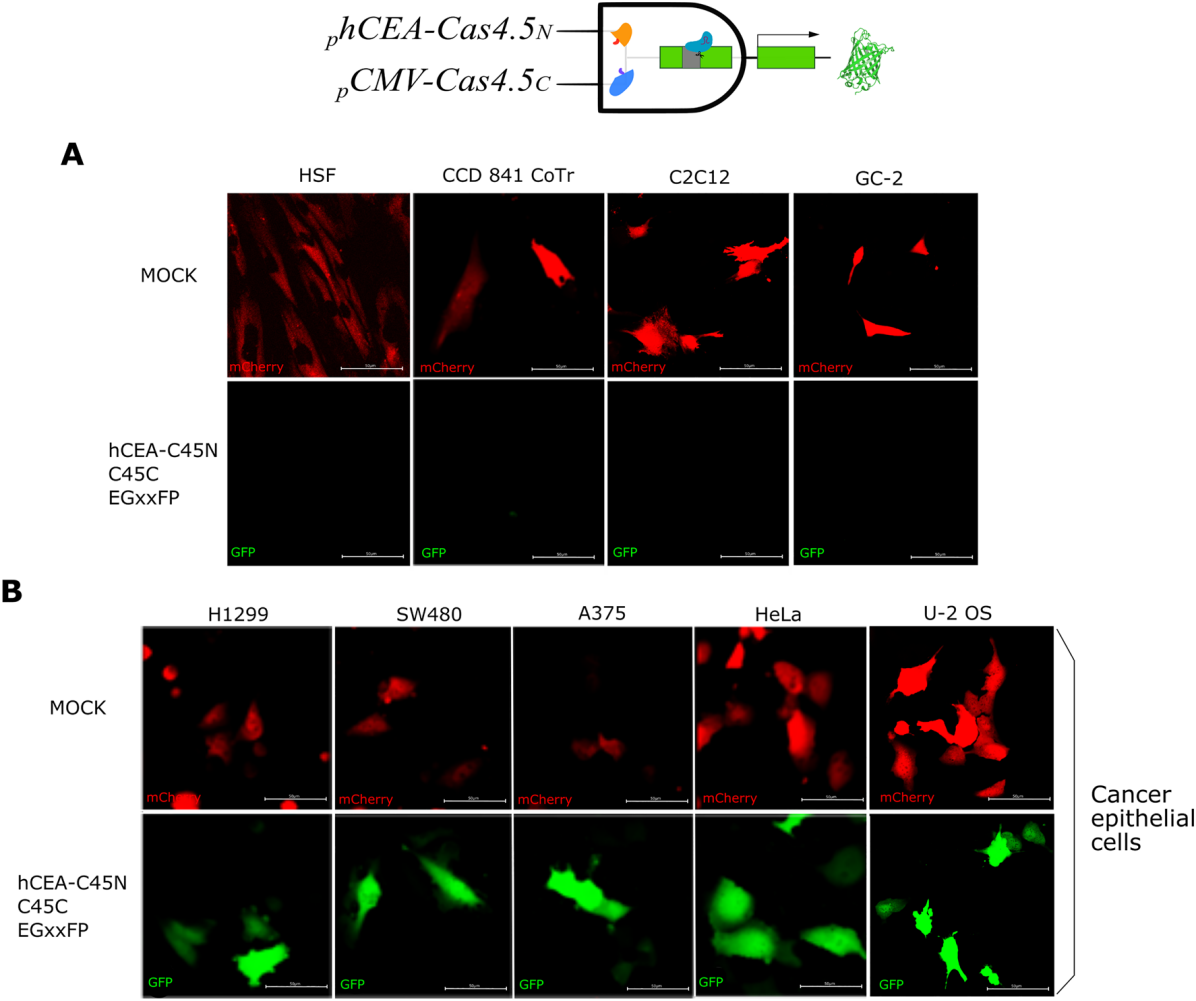
Logic gate to identify only cancer cells of epithelial origin using halves of split Cas9 under a universal (*pCMV*) and a cell type-specific (*phCEA*) promoters. The expression of the green fluorescent protein reporter is observed only in cancer cells of epithelial origin. All cells were co-transfected with _*p*_*hCEA-Cas4.5*_*N*_, *pCMV-Cas4.5*_*C*_/*gRNA*, the EGxxFP reporter and *pCMV-mCherry* (transfection control) constructs. Co-expression of the split Cas9 halves resulted in holo-enzyme formation via inteins, followed by Cas9-induced homologous recombination of EGFP. Only cancer cells of epithelial origin expressed both halves since the expression of Cas4.5_N_ is restricted to these cells. Neither non-epithelial cell lines: human skin fibroblasts (HSF), murine myoblasts (C2C12), nor normal (non-cancerous) epithelial cells**:** CCD 841 CoTr and GC-2**,** showed any expression of the reporter (EGFP) (**A**), while all cancer cells with epithelial origin tested (H1299, SW480, A375, HeLa, and U-2 OS cells) expressed EGFP (**B**).

As a proof-of-concept, spilt Cas9 was designed to selectively sense cancer cells of epithelial origin, using an epithelial cancer-specific promoter (_*p*_*hCEA*). Carcinoembryonic antigen (CEA) has been previously used as a cancer marker due to its selective expression^42,43^, and its promoter has been proposed as cancer-specific and therapeutically useful^44^. Based on that we cloned *Cas4.5*_*N*_*-DnaE*_*N*_ under the _*p*_*hCEA*. The logic gate (_*p*_*hCEA-Cas4.5*_*N*_*-DnaE*_*N*_/*pCMV*-*DnaE*_*C*_*-Cas4.5*_*C*_ and gRNA) was then tested on multiple cell lines, and based on their known origins, we expected expression of *EGFP* only in epithelial cancer cells. For this, we selected various non-epithelial cells such as human skin fibroblasts (HSF) and myoblasts (C2C12); also normal epithelial cells (colon CCD 841 CoTr and spermatocyte GC-2), and cancer cells with epithelial origin: namely H1299 human non-small cell lung carcinoma, colon cancer cell line SW480, malignant melanoma cell line A375, cervical carcinoma cell line HeLa, and U-2 OS human bone osteosarcoma cell line. The logic gate resulted in *EGFP* expression in a pattern matching the cell lines’ origins—where only cancer cell lines of an epithelial origin had all elements expressed and thus *EGFP* expression, which means that all elements have been expressed and once the holo-Cas9 is formed it efficiently activates the reporter. The results confirmed selective expression _*p*_*hCEA*-driven construct **(**Fig. 2). Our results are consistent with the known origins of the studied cell lines. Neither the non-epithelial cells (HSF and C212) nor the non-cancer epithelial cells (CCD 841 CoTr and GC-2) expressed the reporter, despite their comparable transfection efficiency to other lines (mock control) (Fig. 2A). In contrast, all cancer cells with epithelial origin (H1299, SW480, A375, HeLa, and U-2 OS) expressed the reporter (EGFP) (Fig. 2B).

**Figure 3:**
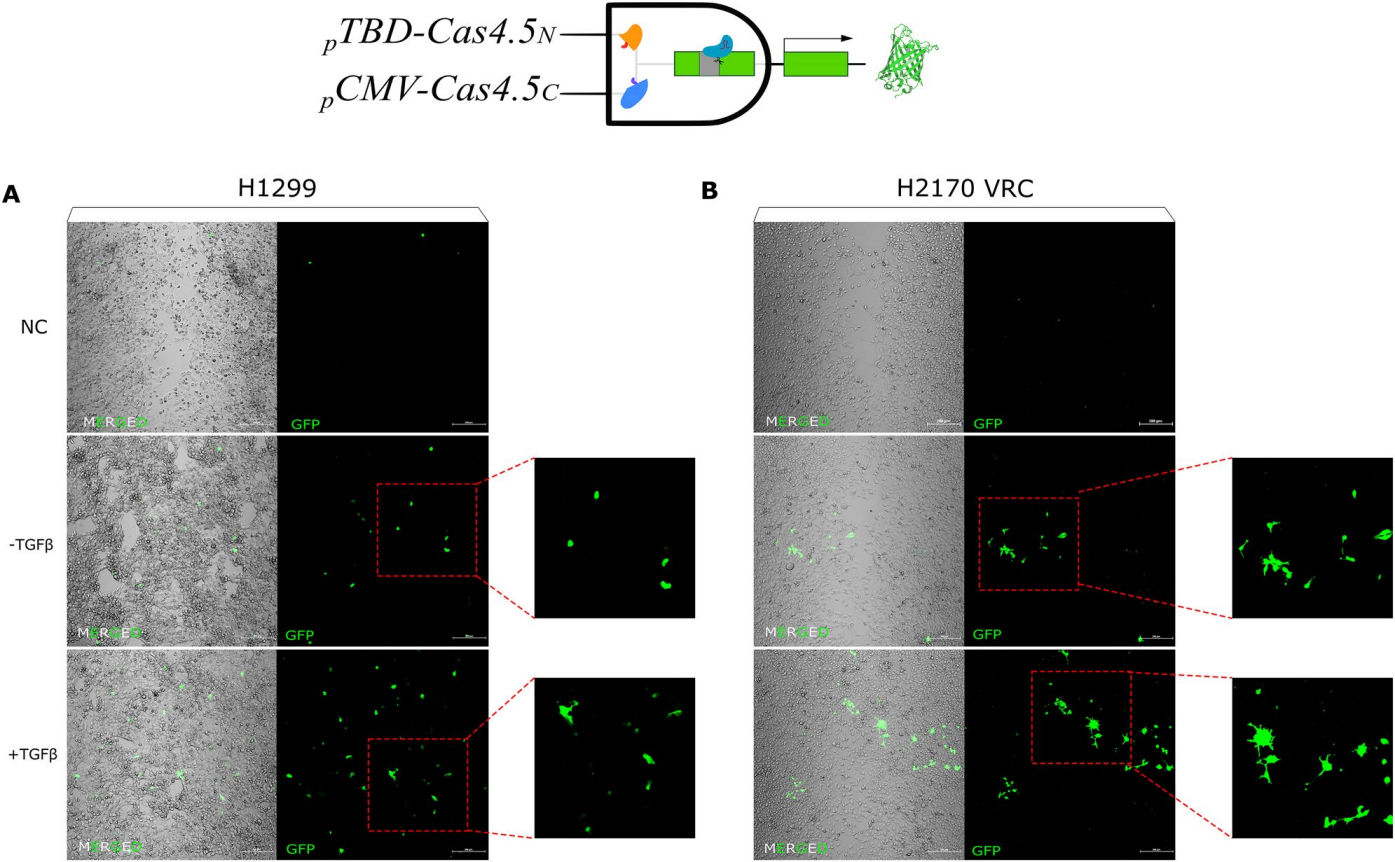
Logic gate for sensing cells that are undergoing EMT. Representative images of H1299 lung cancer cells (**A**) and H2170 VRCs cells (**B**), both partly mesenchymal. Both cell lines, when exposed to TGFβ (+ TGFβ) as an EMT enhancer, increased the number of *EGFP* expressing cells as a proof of undergoing EMT. Morphological changes were visible after activation of TGFβ showed features of cells of mesenchymal origin—more spindle-shaped, they had more protrusions facilitating migration in comparison to controls.

Recognising that a cell type can be labelled based on its gene expression characteristics, we decided to create another logic gate where a biological process is sensed—in this particular case, epithelial-mesenchymal transition (EMT). For this, we exchanged the universal promoter (_*p*_*CMV*) before *Cas4.5*_*C*_*-DnaE*_*C*_ to a promoter that is activated by one of the essential EMT factors, TWIST1^45,46^. TWIST1 activity is closely associated with EMT, correlating with tumor growth, metastasis and drug resistance, thereby diminishing the survival of cancer patients^47^. Using an artificial promoter containing TWIST1 binding domains (*TWIST1-BD*)^36^, driving one half, while the other half under the *pCMV*. As a result, only cells undergoing EMT, or having a mesenchymal phenotype, will express both Cas4.5 halves. To further trigger EMT, cells were exposed to TGFβ. Using the wound healing assay, 48 h after the addition of TGFβ, we began to observe higher reporter activity in epithelial carcinoma cells, both partially mesenchymal, H1299 and H2170 VRCs cells^39^ **(**Fig. 3A,B), predominantly at the edge of the wound, as it is often reported^48,49^. The cell morphology, the number of protrusions, on cells that transitioned into a more mesenchymal phenotype are clearly visible thanks to the reporter (Fig. 3B). While both these lines (H1299 and H2170 VRCs) are known to exhibit partial mesenchymal characteristics, and thus some reporter cells being observed in the non-induced controls (-TGFβ), the number of reporter-expressing cells increased dramatically after EMT induction, accompanied by noticeable changes on phenotype—more protrusions (insets in Fig. 3A,B).

**Figure 4:**
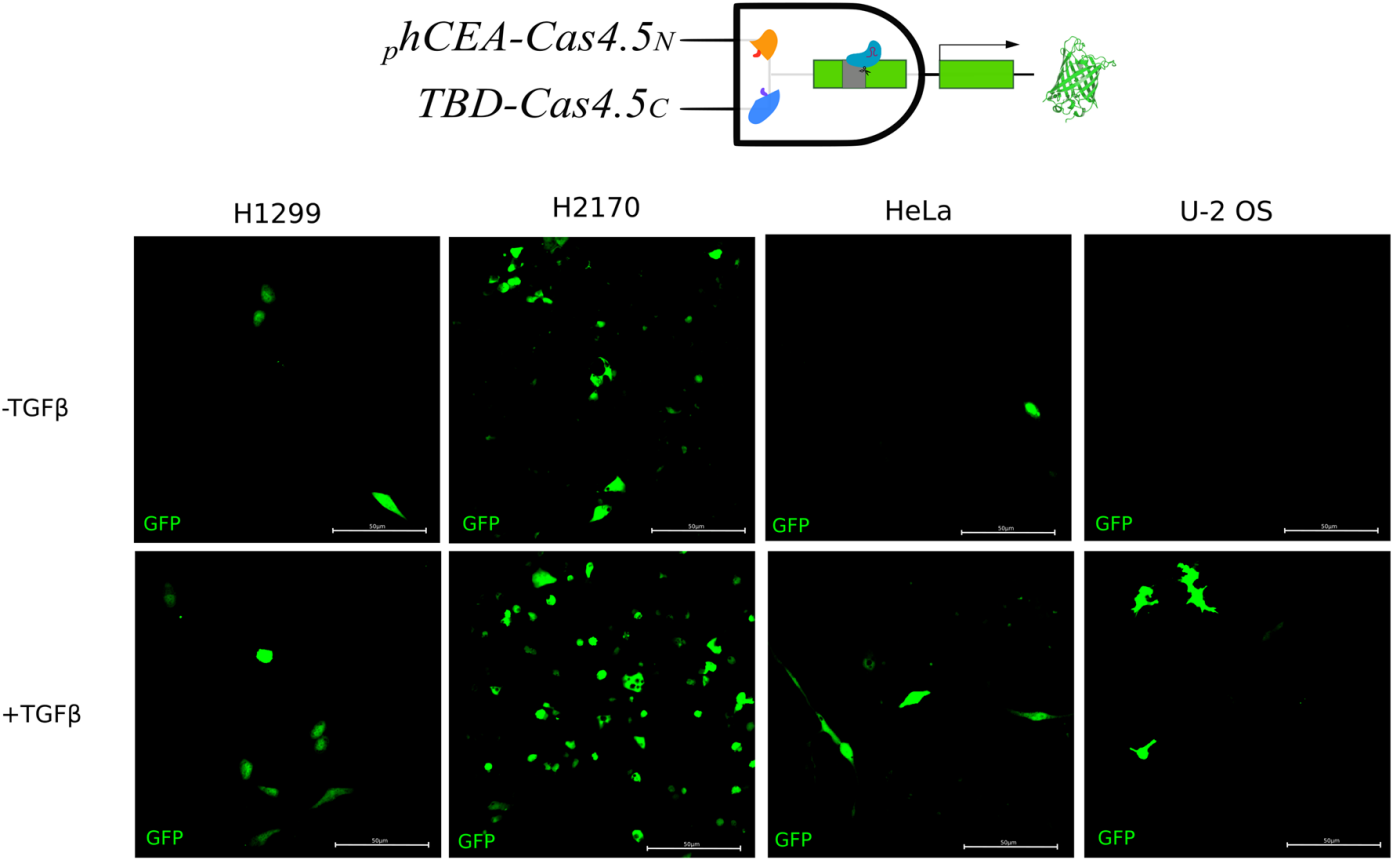
A two level AND logic gate, where epithelial cancer cells undergoing EMT are detected. Four epithelial cancer cell lines were transfected with split Cas9/gRNA system and the EGxxFP reporter. Then cells were stimulated to undergo EMT by the addition of TGFβ. While all these epithelial cancer cells were known to express the *phCEA* (Fig. 2), only cells with partial-EMT expressed the *pTBD*-Cas4,5_C_, without EMT stimulation (upper row, -TGFβ). But when EMT was induced (+ TGFβ), the number of positive cells increased dramatically in those partially mesenchymal (H1299 and H2170), and appeared in those more epithelial—HeLa and U-2 OS cells (lower row).

In order to create a true logic gate, where both inputs are conditional, we designed an AND logic gate consisting of one half of Cas9 under the p*TWIST1-BD*, while the other half under the _*p*_*hCEA* promoter. This configuration should selectively function only in cancerous cells with epithelial origin that are undergoing EMT. Following transfection with all plasmids into cells, they were exposed to EMT activator TGFβ. In the negative control, where no TGFβ was added (-TGFβ), only cancer epithelial cells partly mesenchymal (H1299, H2170) resulted in a lower number of cells expressing the green fluorescent reporter protein, while phenotypical epithelial cells (HeLa and U-2 OS) showed almost no reporter signal. However, upon exposure to TGFβ (+ TGFβ) the number of EGFP-expressing cells increased in every cell line tested (Fig. 4).

### Detection of cell–cell fusion events

In addition of changes of gene expression in biological processes, there are other processes where two inputs could be detected by split Cas9 logic gate, such as cell fusion. This is both a physiological, e.g., placentation and skeletal muscle formation, and pathological as in cancer^50,51^, viral HIV^52,53^ or CoV2 infections^54,55^.

To detect cell–cell fusion, two groups of cells, were transfected separately with a different halve of Cas9, plus the gRNA and reporter. The logic gate here is that the inputs, *N*- or *C*-Cas4.5, can only be activated when two different cells fuse to each other, sharing a common cytoplasm.

To induce fusion, we applied two methods: the overexpression of a “fusogene” Syncytin1—a protein involved in syncytium formation in placenta^56^ that is known to promote the formation of multinucleated cells (syncytia)^57,58^; or a chemical-based method where polyethylene glycol (PEG), due to its known fusogenic properties^59^.

**Figure 5:**
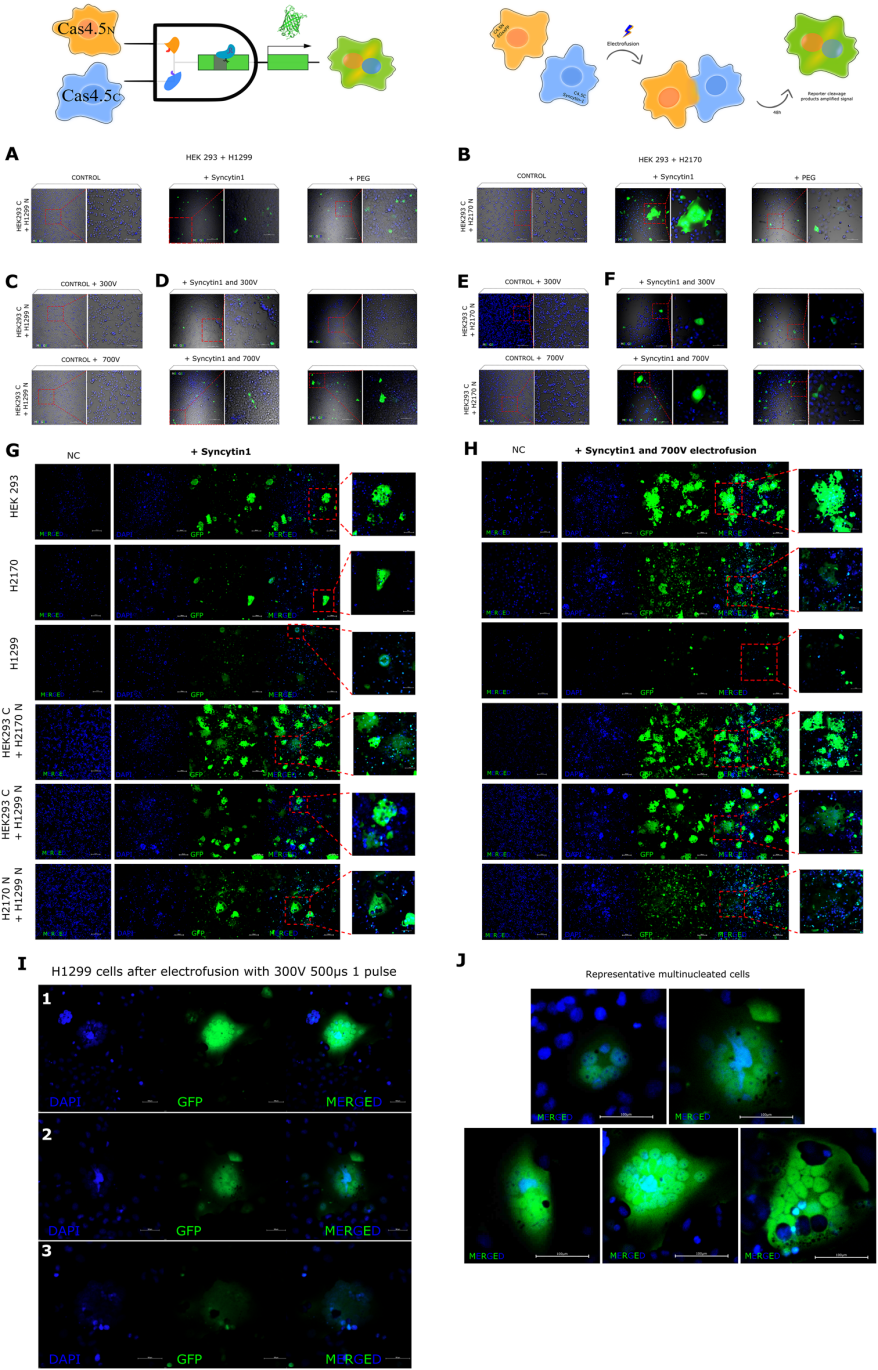
Logic gate to detect cell–cell fusion and schematic diagram of cell–cell electrofusion and hybrid cell formation. Images of admixed transiently transfected cells with complementing Cas9 halves HEK 293 + H1299 and HEK 293 + H2170 after fusion induction by overexpression of Syncytin-1, alone or in pair with electrofusion, or by PEG treatment. Control cells (NC) were transfected with only one half of Cas9 plus the reporter and fusogenic vectors (**A**,**B**). As we expected admixed cells HEK293 + H1299 expressing either one halve (as a control samples) and electrofused using 300V and 700V do not show any fusion events (**C**), compared to electrofusion of cells expressing both halves where we got results of a few cell fusion (**D**). After enhancement of fusion by electric pulses in pair with Syncytin1 overexpression, we visibly increased occurrence of fusion events (**E**). In control samples of admixed HEK293 + H2170 cells after electrofusion using 300 V and 700 V, also the fusion was undetectable (**F**), in comparison to Syncytin1-induced fusion cells plus electrofusion, where fusion cell were clearly visible (**H**). Images of admixed stable cell lines with complementing Cas9 halves (HEK293, H1299, H2170, HEK293 + H1299, HEK293 + H2170, and H1299 + H2170) after fusion induction by overexpression of Syncytin1, and Syncytin1 plus electrofusion. Control cells (NC) were transfected with only one half of Cas9 plus the reporter and fusogenic vectors. In relation to cells only expressed fusogenic Syncytin1 (**G**), the combination of Syncytin1 and electrical pulses (**H**) greatly enhanced the detection of syncytia. When fusing HEK293 with H1299 or H2170, the syncytia visibly formed contained more nuclei, than lung cancer cells in a mix within the same line. In summary, H1299 cells overexpressing Syncytin1 in pair with electrofusion at 700 V did not show significant fusion events (**H**). Higher efficiency was achieved by expression of Syncytin1 and lower voltage (300 V 500 µs one pulse) in stable expressing complementing Cas9 halves H1299 cells (**I**). Representative large multinucleated syncytia after electrofusion cells overexpressed Syncytin1 H1299 (using 300 V), H2170 cell lines (using 700 V), and mixed HEK 293 with H1299 cell line and HEK293 with H2170 cells (using 700 V) (**J**).

For this purpose, we used transiently transfected cell lines (HEK293, H1299, H2170), each line expressing only one of the halves of Cas9 at a time (Cas4.5_C_ or Cas4.5_N_), and co-transfected with the reporter (EGxxFP), as control cells, or either co-transfected with Syncytin1 plasmid or PEG-treated to induce cell–cell fusion. 24 h after transfection, cells expressing one half were admixed with either the same lineage, but expressing the complementing Cas4.5, or with a different cell type but expressing the complementing Cas4.5. As expected, in control samples cell fusion was undetectable, while a number of fusion events occurred, in particular in mixed cells HEK293 + H1299 and HEK293 + H2170, in those either expressing Syncytin1 or PEG-treated (Fig. 5A,B). However, treatment with PEG showed toxic effects on cells and therefore we continued only with Syncytin1 overexpression for further experiments. We then tested electrofusion—the use of high voltage electrical pulses to induce fusions of cells in close proximity^60^—on an admixture of the cells expressing either halve. For this aim, different voltages were tested: 300 V 500 µs one pulse vs 700 V 15 µs three pulses. Compared to control samples, where cell fusion was undetectable (Fig. 5C,F), in cells expressing both halves, electrofusion resulted in a few fusion events (Fig. 5D,G). Thus, to enhance fusion, we then combined both approaches, electrofusion and either expression of Syncytin1 (Fig. 5E,H). As a result, 700 V stimulation together with Syncytin1 induced fusion more effectively than the 300 V, in combination of different cell types HEK293 + H1299 (Fig. 5E), and HEK293 + H2170 (Fig. 5G). We show that in a mix of cells HEK293 + H2170, Syncytin1 overexpression alone and in pair with electrofusion visibly increased fusion events (Fig. 5B,H).

For more in-depth inquiry, we created stable cell lines, and used optimized settings to induce fusion of different cell, each line stably expressing one half of Cas9 (Cas4.5_C_ or Cas4.5_N_), and co-transfected with the reporter (EGxxFP) and Syncytin1 plasmids. 24 h after transfection, the different cell types were admixed within either the same lineage, but expressing the complementing Cas4.5, or to a different cell type also expressing the complementing Cas4.5 (HEK293 with H2170, HEK293 with H1299, and H2170 with H1299) and then cells were electrofused (700 V for 15 µs). Control cells were co-transfected with only one half of Cas9 plus the reporter and Syncytin1 plasmids (negative control, NC). Compared to cells only overexpressing Syncytin1 (Fig. 5G), the presence of Syncytin1 and electrical pulses (Fig. 5H) greatly enhanced the detection of syncytia. We show that in a mix of different type of cells (when fusing HEK293 with H1299 or H2170) the syncytia formed were larger and contained more nuclei, than either cell type fused with its same lineage (Fig. 5G,H). There were no major fusion events in the mix of H1299 cells, whether only Syncytin1 was overexpressed (Fig. 5G) or in pair with 700 V stimulation (Fig. 5H). In summary, cells survival was lower after 1 long vs 3 shorter pulses. Surprisingly, the H1299 cells generated more fusion events at lower voltage (300 V for 500 µs) and 1 pulse (Fig. 5I). The presence of Syncytin1 and electrical impulses contributed significantly for syncytia to occur in H1299 (using 300 V) and H2170 cell lines (using 700 V), and mixed HEK 293 with H1299 or HEK293 with H2170 cells (using 700 V) (Fig. 5J).

**Figure 6:**
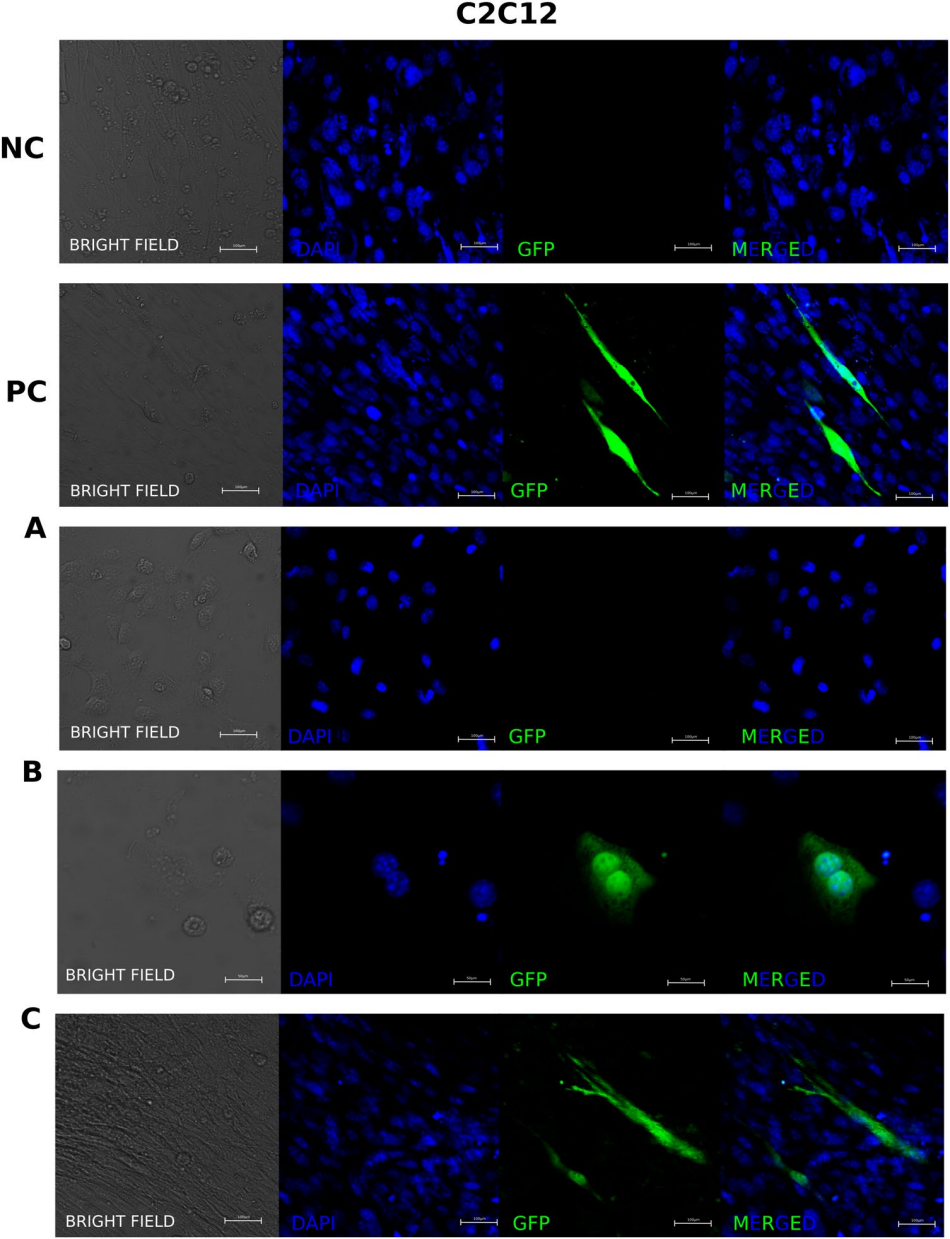
Applying split Cas9 logic gate to detect naturally occurring cell fusions. Murine C2C12 myoblast cells were transfected separately with either Cas9 half (Cas4.5_C_ or Cas4.5_N_), gRNA and EGxxFP reporter, and then co-cultured together in differentiation medium. (**A**) C2C12 cells have a triangular shape while undifferentiated; (**B**) then they align with each other and begin to fuse—for better visualization of the two nuclei this image is show at higher magnification; (**C**) forming myotubules. NC—Negative control (cells with Split Cas9 halves, and EGxxFP reporter without gRNA); PC—(cells transfected with Cas9/gRNA and EGxxFP reporter). Additional images in Supplementary Fig. S1.

We then asked whether we could detect naturally occurring cell fusions. For this, we selected murine C2C12 myoblast cells (Fig. 6), which undergo spontaneous fusion during differentiation into myotubules. C2C12 cells were separately transfected with either Cas9 half (Cas4.5_C_ or Cas4.5_N_), gRNA and EGxxFP reporter. 24 h hours after transfection, cells were trypsynised and co-plated at 90% confluence. Subsequently, differentiation medium was added, and cells were incubated for up to seven days.

As a positive control, we used cells transfected with Cas9, gRNA and EGxxFP reporter. As a negative control, we employed cells transfected with Split Cas9 halves and EGxxFP reporter without gRNA.

Undifferentiated C2C12 cells have a triangular shape (Fig. 6A), but as they differentiated they became spindle-shape, aligned to each other and fused to each other (Fig. 6B), eventually forming myotubules (Fig. 6C).

## Discussion

Prokaryotes, which include bacteria and archaea, have simpler signaling processes than eukaryotic cells, which means that SynBio logic gates are easier to generate. In mammalian systems signaling is rather complex and therefore simple SynBio circuits are more demanding to design.

While biological processes or events can be detected using biochemical methods, the use of bioassays have become more and more commonplace. Yet, many bioassays contain single inputs and can hardly be adapted to other purposes. Here, we show that it is possible to generate logic gates using a split CRISPR/Cas9, which is an actuator with many downstream options^61–64^. Moreover, the activation of Cas9 also creates a memory so using this system we could detect biological processes after they occurred e.g. in vivo long after they occurred.

As CRISPR-Cas-mediated genome editing technologies have provided accessible and flexible means to alter, regulate and visualize genomes, they are considered a milestone for molecular biology in the twenty-first century. So far, CRISPR-Cas systems have found wide applications in the investigation of gene function, gene therapy, development of targeted drugs, and construction of animal models, which fully proves their great potential for further development^10,65–68^.

Together with other synthetic systems for detecting biological phenomena such as optogenetic, gene expression and cell-free sensors, CRISPR systems have shown incredible flexibility that can be easily translated to various research and medical contexts. For example, Liu et al. reported a modular AND logic gate based on the CRISPR-Cas9 system, contributing to a synthetic biology platform for targeting and controlling bladder cancer cells in vitro^27^.

Here, we introduced a new application of the CRISPR/Cas9, as a sensor for detection cancer cells with epithelial origin, using an epithelial cancer-specific promoter (_*p*_*hCEA*), a biological process—cells undergoing EMT. The selection of the *phCEA*, a marker to detect the presence of cancer cells of epithelial origin is specific for various types of cancer including colorectal cancer^44,69^, supports the concept that CEA is one of the most promising target antigens for colorectal cancer immunotherapy in use. CEA-targeted immunotherapies are presently in the preclinical or clinical phase, including CEA vaccines and CEA-targeted CAR T cells or TCR-modified T cells^70^.

Finally, split Cas9 was used for the detection of cell–cell fusions. A process that has been reported to occur between tumor cells and surrounding cells^71–73^. Where normal cells surrounding cancer cells can acquire malignant characteristics, according to the proverb „lie down with dogs, rise up with fleas”. One of the mechanisms facilitating tumor progression as the tumor spreads to surrounding tissue is cell–cell fusion^74^. Cell–cell fusion has been suggested as one possible mechanism for tumor progression^75,76^. Given that EMT is closely related to tumor invasiveness, there is likely to be a link between cell fusion and tumor invasiveness. When cancer cells fuse with healthy differentiated cells, the resulting hybrid cell can develop the ability to divide uncontrollably and form tumours^77–79^. The most compelling evidence that cell–cell fusion drives cancerogenesis is the report by Lazova and collaborators^80^ where they found that a metastatic melanoma was a hybrid between the patient’s cells and the ones from his brother after an allogeneic bone-marrow transplantation. Since then, more reports of cell–cell fusion show that such events initiate or fuel tumorigenesis^79,81^.

The applications presented above are additional tools to the CRISPR palette. By modifying the genetic elements that control the expression of its individual elements, it can be programmed and created more complex logic gates. One of the possibilities offered by this system is the elimination of a selected cell subpopulation from a given cell mixture through the use of gRNAs directed against essential genes. Thus, the proposed method may have potential for cancer diagnosis, therapy and treatment monitoring.

**Figure 7:**
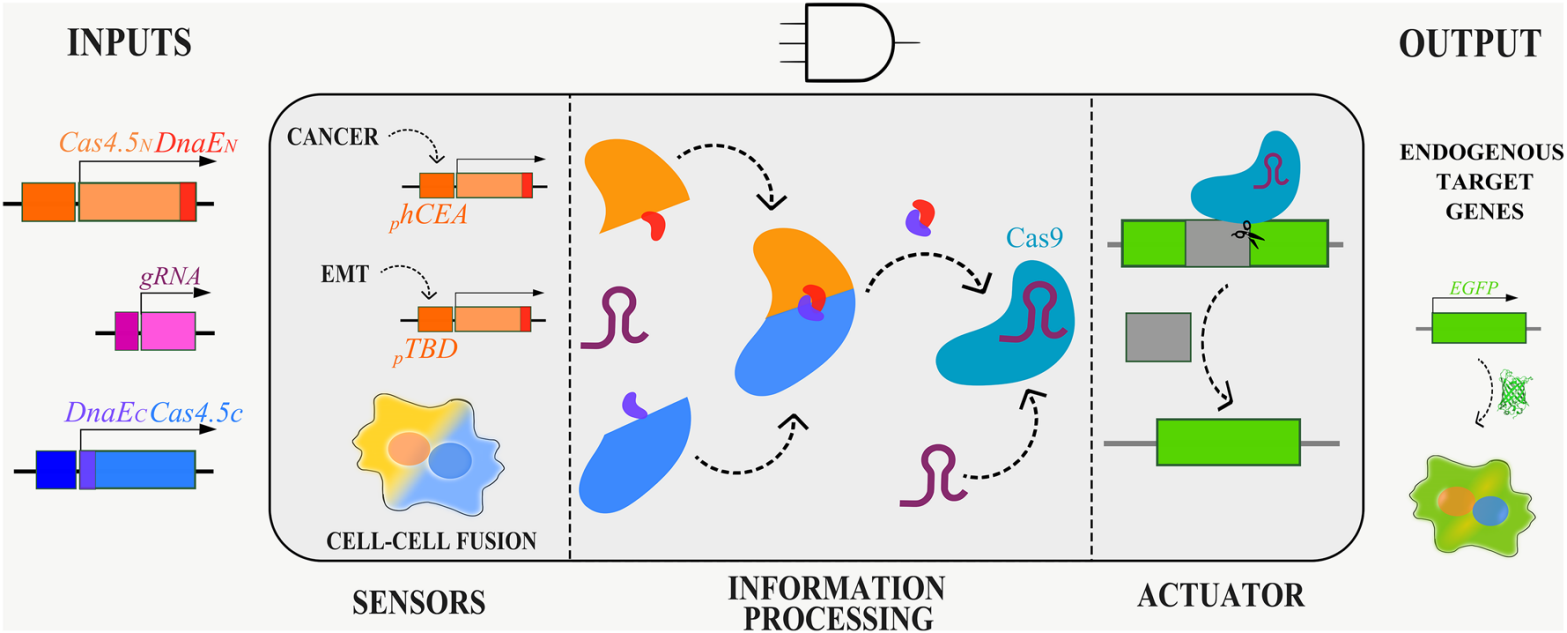
Engineering cells with CRISPR/Cas9 circuits.

In recent years, the potential of using Cas9 as an actuator has also become apparent, is still a relatively new approach, but it has the potential to enable new possibilities for precise control over biological systems. Moreover, the requirement of the gRNA allows a 3-input system that can target specific genomic loci or additional logic gates/circuits, and thus become a research tool for multiple applications (Fig. 7).

In the last few years, SynBio has made evident that it can use engineering logic to provide insight into previously unrecognized mechanisms of natural phenomena^6,82^. As Cas9 has recently been shown to be associated with non-canonical RNAs in bacteria^82^, exploring the interplay among endogenous RNA and Cas9 in eukaryotes could point to new technology, including additional imaging reporters, to study gene activation and even enables therapy modalities in mammalian systems.

In the grand scheme, CRISPR provides new opportunities for constructing circuits that can control processes in living cells, with wide application in synthetic biology. Yet, there are still limitations that need to be overcome, such as improving delivery methods, what could be achieved by utilizing viral vectors or nanoparticles, in especially to primary cells and in vivo conditions. Another limitation lies in the characterisation of promoters or antigens that are only expressed in certain cell types or conditions, which would significantly improve specificity. Despite these challenges, our findings have expanded the use of CRISPR technologies, and we envision the development of more complex gates in the near future.

### Supplementary Information

Supplementary Information.

## Data Availability

All data generated or analysed during this study are included in this published article [and its supplementary information files]. The nucleotide (plasmid) data for this study have been deposited in the European Nucleotide Archive (ENA) at EMBL-EBI under accession number PRJEB64624 (https://www.ebi.ac.uk/ena/browser/view/PRJEB64624.
